# Understanding and defining bullying – adolescents’ own views

**DOI:** 10.1186/2049-3258-73-4

**Published:** 2015-02-02

**Authors:** Lisa Hellström, Louise Persson, Curt Hagquist

**Affiliations:** Centre for Research on Child and Adolescent Mental Health, Karlstad University, Karlstad, SE 651 88 Sweden

**Keywords:** Adolescents, Bullying, Definition, Qualitative content analysis

## Abstract

**Background:**

The negative consequences of peer-victimization on children and adolescents are major public health concerns which have been subjected to extensive research. Given all efforts made to analyze and estimate the social and health consequences of peer-victimization, the adolescents’ own experiences and understandings have had surprisingly little impact on the definition of bullying. Therefore, the aim of the current study is to explore adolescents’ definitions of bullying.

**Methods:**

A questionnaire study (n = 128) and four focus group interviews (n = 21) were conducted among students aged 13 and 15. First, gender and age differences were analyzed with respect to what behaviors are considered bullying (questionnaire data). Second, analysis of what bullying is (focus group interviews) was conducted using qualitative content analysis.

**Results:**

The adolescents own understanding and definition of bullying didn’t just include the traditional criteria of repetition and power imbalance, but also a criterion based on the health consequences of bullying. The results showed that a single but hurtful or harmful incident also could be considered bullying irrespective of whether the traditional criteria were fulfilled or not. Further, girls and older students had a more inclusive view of bullying and reported more types of behaviors as bullying compared to boys and younger students.

**Conclusions:**

The results of the current study adds to the existing literature by showing that adolescents consider the victim’s experience of hurt and harm as a criterion for defining bullying and not only as consequences of bullying. This may be of special relevance for the identification and classification of bullying incidents on the internet where devastating consequences have been reported from single incidents and the use of the traditional criteria of intent, repetition and power imbalance may not be as relevant as for traditional bullying. It implies that the traditional criteria included in most definitions of bullying may not fully reflect adolescents’ understanding and definition of bullying. Assessments of bullying behaviors that ask adolescents to strictly adhere to the traditional definition of bullying might not identify all adolescents experiencing peer victimization and therefore not provide estimates of prevalence rates reflecting adolescents’ own understanding of bullying.

## Background

Given the extensive research and efforts made to estimate the negative effects of peer-victimization, adolescents’ own experiences and understandings have had surprisingly little impact on the definition of bullying. New forms of peer-victimization over the internet, reflecting the changing social conditions among youth today may have altered adolescents’ and children’s views of what behaviors constitute bullying which may challenge previous definitions [1, 2]. Youth’s judgment of what is considered unacceptable behavior (such as bullying) may in some sense be influenced by the exploitation of hurtful and humiliating behavior portrayed on television and on the internet [3–5]. A persistent problem in bullying research is to decide where teasing ends and bullying begins [6]. The intent may be even harder to interpret in non-face-to-face situations over the internet. Given that prevalence rates are critical for planning treatment and prevention [7], it is of great importance to have measurement instruments including definitions that correctly reflect peer relations among today’s youth and that capture the entire phenomenon of bullying.

### Definition of bullying

The most commonly used definitions of bullying are formulated by adults and researchers and state that bullying is intentional, repetitive aggressive behaviors including some sort of power imbalance between those involved [8]. Even if the rationale behind the criteria is to separate harmful behaviors from less harmful behaviors [9], distinctions among different forms of peer-victimization need more empirical foundation [10]. Power imbalance and intention are used as criteria to separate bullying from other forms of aggressive behavior, but have proven hard to operationalize and capture in assessments among children [11–13]. While repetition may be easier to operationalize and measure no generally accepted cut point for bullying exists [14, 15].

### Previous research

Studies have shown that children rarely include the traditional criteria of intent, repetition and power imbalance when defining bullying [7, 16–19]. Girls tend to omit the traditional criteria and mention the effect on the target more often compared to boys [18–20]. In addition, younger children tend to report physical aggression as bullying more often, while older children more often report verbal aggression and social exclusion as examples of bullying [6, 16, 21]. Despite the acknowledgement that children may hold a different understanding of bullying compared to those researching the problem, children’s own view have had little or no impact on the definition of bullying. Rather, suggestions for solving these inconsistencies include adjusting children’s definitions to better coincide with researcher’s definitions [18, 22, 23].

### The current study

Among the studies exploring children’s views on bullying, only few have been conducted with the purpose to take children’s understandings into account when it comes to defining bullying. In addition, many of the earlier studies did not pick up on the different forms of cyberbullying that have increased exponentially in recent years, which justifies a re-examination and validation for the future. A few studies have specifically focused on children’s definitions of cyberbullying [1, 24], viewing it as a separate phenomenon compared to traditional forms of bullying. However, research has shown that negative incidents online are also linked to real-world antisocial behaviors [25, 26] and it has been suggested that traditional bullying and cyberbullying are rather two sides of the same coin [27, 28]. For this reason, the current study did not seek to distinguish between traditional and cyber forms of bullying. For a wider comprehension, the current study will explore adolescents’ definitions of bullying using both quantitative questionnaire data and qualitative data from focus group interviews. While quantitative methodology provides opportunities to make comparisons between different groups, the use of qualitative methodology offers possibilities to develop a deeper understanding of the culture and group processes involved in bullying [29]. The aim of the current study is to explore adolescents’ definitions of bullying.

## Method

### Participants

This study is based on data collected in the spring of 2012 as part of a large project aimed at promoting mental health among schoolchildren (The Preventive School project). The study involved students in the ages 13 and 15 (Grades 7 and 9) from two schools. 128 students (60.9% girls) completed a web-based questionnaire and 21 students (8 girls and 13 boys) participated in four focus group interviews, with separate groups for girls and boys. Each group consisted of members from the same school and of the same school year. In school A students in Grade 7 participated in focus group interviews while students in Grade 9 participated in the questionnaire. In school B students in Grade 7 participated in the questionnaire while students in Grade 9 participated in focus group interviews.

### Procedure

**Figure 1:**
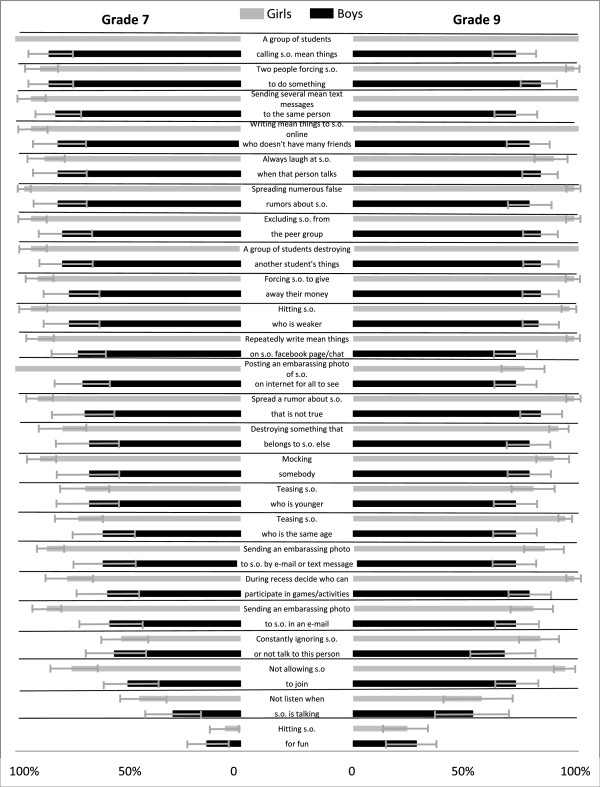
**Behaviors reported as bullying (%) among girls and boys participating in The Preventive School project in Sweden 2012: results for adolescents in Grade 7 presented at the left and Grade 9 presented at the right (95% C.I.).** [Note: s.o. is used as an abbreviation for someone].

Two schools were selected to be included in a web-based questionnaire study and a focus group study after agreement from the responsible principals. The schools were chosen because their large size was expected to provide a great variety and selection of students. First, the principals were each told to select and invite three classes to participate in the questionnaire study. A questionnaire was designed and consisted of 24 behavior descriptions depicting varying conditions based on questions of bullying behaviors used in Olweus Bully/Victim Questionnaire (OBVQ) [30]. The questions included the specific forms of bullying asked for in OBVQ with alternating use of the three bullying criteria intent, repetition and power imbalance. That is, the questions included none or any of the criteria for bullying (see Figure 1). The students were asked to answer whether they considered the behaviors to be bullying or not with a “yes” or “no” answer. The students were also given the opportunity to comment on their responses in an open-ended format. A researcher was on site when the students completed the questionnaire to answer potential questions. For the questionnaire study and the focus group study, the students and parents were given written information in advance and the students were informed that their participation was voluntary, that their answers were anonymous, and that they could terminate their participation at any point. The parents of the students in Grade 7 were asked to sign a written consent for their child’s participation in the study. For students in Grade 9, parental consent was not required.

Second, all students in Grade 7 and 9 were asked to contact their class teacher if they wanted to participate in a focus group interview. The students who volunteered first were invited to participate. Four same-gender and same-age groups of 4–7 students were arranged and the interviews took place at the students’ schools and lasted about an hour. The students were orally informed that they could choose to refrain from talking about any specific topic during the interview and they agreed to recording of the interview in writing. The interviewer [i.e. the first author] conducted the focus group interviews and a research colleague [public health researcher] assisted with follow-up questions and questions of clarification. The question of interest in the focus group interviews were “What do you think bullying is?” and was followed up by questions such as “can you develop what you just said”, “what do you mean” and “can you give any examples”. Before the focus group interviews ended the students were asked if they had anything to add or if they thought that something important had been left out of the discussion. Each focus group interview was transcribed verbatim.

### Analysis

First, different types of behavior that the adolescents considered to be bullying (questionnaire data) are reported. The differences in perceived bullying behaviors between boys and girls are tested among Grade 7 and Grade 9 students using Chi square statistics. In total, 48 significance tests were performed. Therefore, the Bonferroni adjustment [31] was applied in order to adjust for the influence of multiple significance tests. This implies that the significance level for significant differences between girls and boys in Grade 7 and Grade 9 was set to 0.05/48 = 0.0010. Second, data analysis of the focus group interviews was conducted using qualitative content analysis [32]. Descriptions of what bullying is constituted the unit of analysis. First, the transcription of each focus group interview was read through several times to get a sense of the material. Second, meaning-carrying units which responded to the aim of the study were extracted. Third, the meaning-carrying units were condensed and abstracted into codes. In order to identify similarities and differences the codes were compared and then sorted into sub-categories (Table 1). As the analysis proceeded, subcategories were subsequently clarified and adjusted and one main category emerged. The initial coding of the transcripts was performed by the first author, and the coded data were examined by the second and third author for emergent sub-categories. The interpretations were compared and discussed until consensus was reached. Comparisons were made with the context in each step of the analysis, to verify the empirical base of the data. The pupils answered in Swedish and the quotations cited were translated into English after the analysis.

**Table 1:** **Qualitative content analysis of what adolescents in The Preventive School project (Sweden, 2012) think bullying is**

Meaning-carrying unit	Condensed meaning-carrying unit	Code	Sub-category	Category
… I believe that bullying is more like… you can only know yourself if you have been bullied or not, I think. Because you have… everybody think differently about what bullying is.	One can only know yourself if you are being bullied or not because everyone thinks differently about what bullying is	Only you can know if you’ve been bullied	Self-interpretation	The core of bullying
I may think it is bullying but someone else may not think it’s bullying. So it could be really different.	I may think that something is bullying but someone else does not think it’s bullying	Think differently about what bullying is.	Self-interpretation	The core of bullying
It is one person being oppressed by the other. So one who feels weaker. Maybe does not dare to say what he or she thinks. And then the other oppresses that person. Then it’s more like bullying	One who becomes oppressed and feels weaker. Does not dare say what he or she thinks	A weaker person being oppressed	Behavior descriptions	The core of bullying
If you post a picture and someone writes ugly … well the one who becomes a victim of bullying posts a picture, and the other says ugly things … well, writes ugly things … like comments to the picture	You post a picture and someone writes ugly comments to the picture	Write ugly comments to pictures	Behavior descriptions	The core of bullying

## Results

Figure 1 report results from the questionnaire study regarding adolescents’ perception of what types of behaviors they considered as bullying. Chi square tests were performed to analyze gender and grade differences. Among Grade 9 students, significantly more girls compared to boys reported the following behaviors to be bullying: ‘repeatedly write mean things on someone’s facebook page or in a chat’ (p ≤ 0.001), ‘sending several mean text messages to the same person’ (p ≤ 0.001), ‘a group of students calling someone mean things’ (p ≤ 0.001) and ‘writing mean things to someone online who does not have many friends (p ≤ 0.001). Similar results were found among Grade 7 students. While ‘hitting someone for fun’ was reported as bullying twice as often among boys in Grade 7 (15%) compared to girls in Grade 7 (7%), the differences were non-significant. The results revealed that in general, students in Grade 9 more often reported the different behaviors as bullying compared to students in Grade 7. Students in Grade 9 reported behaviors such as social exclusion to be bullying more often compared to students in Grade 7, e.g., ‘constantly ignoring someone or not talk to this person’, and ‘during recess decide who can participate (or not participate) in games or other activities’. Comments regarding their responses included circumstances under which the adolescents were more likely to consider the behaviors as bullying, namely; the effect on the victim (e.g., ‘I think it’s bullying when the person being exposed think it’s bullying… If it’s for fun it doesn’t have to be bullying, as long as no one feels bullied’); if both parties are in on it (e.g., ‘posting an embarrassing photo can be okay, if the person is in on it, or if it’s posted in a Facebook-group where similar photos are posted’); repetition (e.g., ‘much of it is bullying but it depends if the behavior is repeated’); and intent (‘it depends whether you mean it or not’).

One main category and three sub-categories emerged from the analysis of the focus group interviews. The main category was: ‘The core of bullying’.

### The core of bullying

The core of bullying includes different aspects that the adolescents used to describe what bullying is, and consists of three sub-categories; behavior descriptions, self-interpretation, and something hurtful.

#### Behavior descriptions

According to the adolescents, bullying behavior included; teasing, giving nasty comments, fussing, oppression or threats with words. Often, comments were used as a way to oppress someone else and consisted of jokes about where you come from, your clothes or the way you look. Boys were perceived as more straightforward in their comments ‘they [boys] are frank…they can say ‘what an ugly hat you have’. But a girl wouldn’t do that, she would whisper it’ [Girl, age 13]. It was expressed that bullying behaviors such as hitting, pushing or tackling someone were more common among boys and that they often egg on each other to retaliate and to not back down when they are in arguments. Expectations from adults were also mentioned as a possible explanation for gender differences in bullying behavior;

‘Among teachers and grown-ups for example, if it’s boys fighting or if it would be some girls fighting physically… I mean, it’s not as acceptable. Therefore, it is easier to take it verbally. Instead of…or you will be judged somehow…a thousand times just because you hit someone. Not a lot of girls fight physically…’ [Girl, age 15].

Other bullying behaviors mentioned by the adolescents were talking behind someone’s back, whisper and looking down on someone, spreading rumors, giving glances, ignoring, avoiding, or ejecting someone from the group. Bullying also included malicious behavior for example posting pictures and mean comments on social media sites such as Facebook and Twitter. Repeated jokes could also turn into bullying within the peer-group. Recurrent events happening over a long time-period were described as essential for defining bullying behaviors ‘Because they are joking… but if they repeat it, it automatically becomes bullying I think’ [Girl, age 15]. The adolescents pointed out that in contrast to bullying, occasional arguments or fights were solved right away and all involved had an equal share in the argument and in the chance of “winning”. Bullying was further described as behaviors involving a group against a single individual or as quarrel between two persons where one had difficulty standing up for himself or herself.

#### Self-interpretation

It emerged that determining the circumstances for when a behavior should be considered bullying was very much a question of self-interpretation. It was expressed that not being able to interpret the tone of voice or facial expression made it harder to separate jokes from bullying, especially over the internet. The adolescents further mentioned that when someone takes offense and feels bad as a consequence the incident should be considered bullying. Even if it just happens once and even if it was meant as a joke ‘I think the line should be drawn when someone stops laughing’ [Boy, age 13]. However, this boundary could be different for different people ‘I mean, I think it’s hard to know what bullying is. That’s why I think that you are the only one who can decide whether you’ve been bullied or not. Because…everyone thinks differently so it’s really hard to know’ [Girl, age 15]. It emerged that if you knowingly bully someone, some adolescents considered it to be bullying even if the person did not get offended while some adolescents argued that the victim has to be offended for it to be considered bullying.

#### Something hurtful

The adolescents described bullying as something hurtful that leads to negative health consequences. Verbal bullying in particular leaves scares that lead to low self-esteem and feelings of not being good enough. Being different and standing out could mean that no one wants to be with you and that you sometimes have to stand the bullying in order not to be alone ‘If you really get bullied, I mean real bullying than maybe…you shouldn’t even be with them. But otherwise you have to walk around alone’ [Girl, age 13]. It was expressed that bullying leads to sadness, especially if you are bullied due to reasons you cannot change and if no one backs you up. Bullying taking place both at school and on the internet were seen as particularly hurtful. According to the adolescents, the comfort of hiding behind a computer screen often made bullying incidents online more aggressive and rawer compared to bullying in real life. Despite this, the adolescents meant that incidents online were easier to dismiss ‘It’s easier to keep your distance on the internet. It’s easier to ignore. I don’t think that you take it as serious…there are more ways to remove that person from your life…like Facebook, you can just block someone…you can’t do that in real life’ [Boy, age 15].

## Discussion

The current study was conducted to explore adolescents’ definitions of bullying. The questionnaire results show that older students are generally more inclusive when it comes to determining what types of behaviors constitute bullying compared to younger students. The older students reported more types of behaviors as bullying and more often reported behaviors such as social exclusion as bullying compared to the younger students. The present findings are similar to those of others [21, 33, 34] who suggest that children’s understandings of bullying change with age and younger children more often view aggressive behaviors such as fighting as bullying while older students often have a more differentiated understanding of bullying including non-physical behaviors such as verbal aggression and social exclusion in their perception of bullying behaviors. Regarding gender differences, the boys in the current study reported fewer behaviors as bullying in comparison to the girls and a larger proportion of girls compared to boys considered behaviors on the internet and behaviors involving the peer group as bullying. While some researchers have found no gender differences in understanding and defining bullying behavior [16, 21, 33], others have shown that females in general tend to define behaviors as bullying more often and to ascribe more severity to different behaviors and boys tend to classify potential conflicts as harmless horseplay [23, 35]. This could be a conscious coping strategy among boys and could also be an explanation of why boys are more restricted in their responses of what is considered bullying. Recent research has shown that girls tend to be more engaged in bullying online [36], which could explain why girls interpret aggression online as bullying more often compared to boys.

The adolescents in the current study defined and described bullying behavior by including some of traditional criteria included in most definitions of bullying [8], i.e., repetition and power imbalance, while intent was not highly emphasized. There was an agreement among the adolescents that inequality is common in bullying such as one person not being able to defend oneself. Hence, when those involved stood up for themselves it was seen more like common brawl. However, the adolescents found it hard to identify the exact circumstances for when a particular behavior should be considered bullying, especially over the internet. On the one hand, it was described that repeated behaviors, even jokes, was automatically considered bullying. On the other hand, even occasional incidents could be considered bullying if the victimized person felt bad as a consequence, irrespective the intent behind the behavior. The line was drawn when the person stops laughing.

The idea behind including intention, repetition and power imbalance in the definition of bullying is to single out the most harmful behaviors [9]. However, there is some disagreement in the bullying literature whether the effect on the victim is implicitly stated in most definitions of bullying [16–18]. Although previous studies on children’s and adolescents’ definition of bullying have indicated that they often describe bullying as negative behaviors with harmful consequences [7, 22, 37], the results from this study show that adolescents’ also focus on the victim’s feelings to decide whether a behavior should be defined as bullying. Children’s focus on the victim’s experience rather than the bully’s intent has been reported by a few others [16, 17]. In contrast to most research reporting the negative effect on the victim only as a consequence of bullying, the results in the current study show that adolescents also include the negative experience of the victim as a criterion for defining bullying. Since the effect on the victim is judged subjectively, its interpretation may vary greatly due to individual vulnerabilities. Despite this, the established association between distress and peer-victimization may justify an inclusion of the negative effect on the victim as a criterion for bullying [38]. Incidents that could be seen as irrelevant for outsiders may be of major importance for the exposed child [39]. Discrepancies in adults’ and children’s views become problematic if studies on bullying rely on definitions that children are not able to relate to; e.g. the risk for miscommunication and passive responses by adults may increase [34]. The results from the present study highlight problems with traditional definitions of bullying as harmful incidents that are not in line with the stated criteria risk being omitted.

The adolescents in the current study found bullying online to be rawer and more aggressive compared to face-to-face bullying, which is in line with previous research [40, 41]. However, they also considered bullying online to be easier to handle in comparison to incidents happening face-to-face. Even though mean and hurtful behavior may have become normalized in the online communication among adolescents, public incidents online including picture and video sharing may be more hurtful than non-public incidents online [42]. Recent events including beauty contests and public shaming on picture sharing networking sites, highlighted in the Swedish media [43, 44], have increased the understanding of the changing nature of peer relations among today’s youth. Incidents taking place on the internet may have a large negative impact on the lives of the victims, regardless of the fulfillment of the traditional bullying criteria. As the adolescents in the current study put it; what you write on the internet does not go away, it remains there. The objective criteria of intention and power imbalance are harder to interpret over the internet while subjective criteria such as the negative effect and consequences of the incident are easier for the victim to relate to.

As children’s actions are grounded in how they understand and interpret their universe and not in what adults or researchers see as objective reality, students’ perception regarding what is bullying could be the critical missing component in the undertaking of understanding and addressing bullying in schools [35, 45]. Despite every child’s right to express their voice in matters that concerns them [46], children’s views have had little or no impact on the definition of bullying. Based on the results in the current study, and in line with suggestions by other researchers [17, 19], the estimation of bullying prevalence rates need to take children’s perspectives into consideration.

### Methodological considerations

The schools in the questionnaire study were not randomly selected which could limit the representativeness and generalizability of the results. Since the principals were asked to make the class selection, it is possible that the selection of participants may be biased. Using a questionnaire asking adolescents which specific behaviors are considered bullying restricts their judgment of bullying to the given examples. Further, in qualitative research the findings are evaluated in terms of trustworthiness (credibility, dependability, and transferability) [32]. The current study used focus groups to encourage active discussions. The group interaction offered by focus groups encourage people to talk to one another; asking questions, exchanging experiences and commenting on each other’s points of view [47]. In the current study, boys and girls were divided into separate groups to make the group a safe place to discuss bullying [48]. Choosing girls and boys from different schools and grade levels also enhanced the credibility of the data as it offered a richer variation and understanding of the phenomenon of bullying. A broad question on bullying was deliberately chosen to capture adolescents view on both traditional bullying and cyberbullying and to not restrict their answers to one or the other. Further, the trustworthiness was enhanced by involving three researchers in the analysis process to reach consensus and by including quotations from the transcribed text, showing similarities within and differences between categories [32]. The peer dynamics and relations between the participants in the focus groups were not known. Previous negative relations between group members could have impacted on the content of discussions. Adolescents’ perceptions on bullying were identified but the participants were not asked about their personal experiences with bullying. Hence, we do not know whether the participants had been bullied or had bullied others which could have affected their perceptions of bullying. When group members have no personal experience with the topic, their discussions are based on opinions, which questions the transferability of the results in the current study to other groups and contexts [32, 49].

## Conclusions

A good start in the work to prevent bullying is to reach consensus among children and adults concerning what types of behaviors are considered bullying and under what circumstances a behavior should be defined as bullying. All children have the right to express their opinions regarding matters that concerns them and allowing children’s voices to be heard are crucial as they may not always be consistent with adults’ understandings. The results from this study showed that the adolescents own understanding and definition of bullying didn’t just include the traditional criteria of repetition and power imbalance, but also a criterion based on the health consequences of bullying. I.e., a single but hurtful or harmful incident could also be considered bullying irrespective of whether the traditional criteria were fulfilled or not. This adds to the existing literature by showing that adolescents included the victim’s experience of hurt and harm as a criterion for defining bullying and not only as consequences of bullying. This may be of special relevance for the identification and classification of bullying incidents on the internet where devastating consequences have been reported from single incidents and the use of the traditional criteria of intent, repetition and power imbalance may not be as relevant as for traditional bullying. The results imply that the traditional criteria included in most definitions of bullying may not fully reflect adolescents understanding and definition of bullying. Assessments of bullying behaviors that ask adolescents to strictly adhere to the traditional definition of bullying might not identify all adolescents experiencing peer victimization and therefore not provide estimates of prevalence rates reflecting adolescents’ own understanding of what it means to be bullied. Measuring hurt and harm in children is a complex task and raises concern with appropriate cut points. Future research should consider ways to include hurt and harm in peer-victimization assessments.
